# Association of red cell distribution width with all-cause and cardiovascular-specific mortality in African American and white adults: a prospective cohort study

**DOI:** 10.1186/s12967-017-1313-6

**Published:** 2017-10-13

**Authors:** Salman M. Tajuddin, Mike A. Nalls, Alan B. Zonderman, Michele K. Evans

**Affiliations:** 1 Laboratory of Epidemiology and Population Sciences, National Institute on Aging, National Institutes of Health, Room # 04C222, Suite 100, 251 Bayview Boulevard, Baltimore, MD 21224 USA; 2 Laboratory of Neurogenetics, National Institute on Aging, National Institutes of Health, Bethesda, MD 20892 USA; 3 Data Tecnica International LLC, Glen Echo, MD 20812 USA

**Keywords:** Red cell distribution width, Mortality biomarker, Premature mortality, Cardiovascular disease, Survival analysis, African Americans, Whites

## Abstract

**Background:**

While the mortality rate is declining in the United States, the life expectancy gap among different population groups suggests a need to identify biomarkers to improve early identification of individuals at risk. Red cell distribution width (RDW), a measure of anisocytosis, is an emerging biomarker of chronic disease morbidity and mortality, particularly in the elderly. However, little is known about its association with mortality risk in younger adults. The objectives of this study were to investigate the association between RDW and overall and cause-specific mortality risk, and to identify novel determinants of RDW level.

**Methods:**

We used prospectively collected data from the Healthy Aging in Neighborhoods of Diversity across the Life Span study conducted in Baltimore, Maryland. At baseline (2004–2009), the study recruited 3720 African American and white men and women aged 30–64 years. Participants provided peripheral venous blood for RDW measurement as part of complete blood count, and genotyping. Mortality status was ascertained using the National Death Index database through December 31, 2013. Multivariable adjusted Cox proportional hazards regression models were fitted to assess mortality risk, and multiple linear regression models to identify determinants of RDW level.

**Results:**

Participants’ mean age was 48.1 (9.2) years. Of 2726 participants included in the present analyses, 57% were African Americans, and 56% were women. After 18,424 person-years of follow-up time, there were 226 deaths, and the leading cause of death were cardiovascular diseases (31.9%). Participants in the highest quartile of RDW had a 1.73-fold increased all-cause mortality risk (highest quartile vs. lowest quartile, multivariable adjusted hazard ratio = 1.73, 95% confidence interval: 1.10–2.74, p-trend = 0.006). This effect was significantly modified by body mass index (p-interaction = 0.004). Similar risk was observed for cardiovascular disease-specific mortality. Independent of body mass index, waist-hip ratio and illicit drug use were significantly associated with RDW.

**Conclusions:**

Elevated RDW was associated with a substantial risk of all-cause and cardiovascular disease-specific mortalities that was modified by body mass index. Central obesity and illicit drug use influence RDW level. In vulnerable populations at-risk for health disparities, RDW could provide a useful and inexpensive biomarker of mortality.

**Electronic supplementary material:**

The online version of this article (doi:10.1186/s12967-017-1313-6) contains supplementary material, which is available to authorized users.

## Background

Red cell distribution width (RDW), a measure of variation in red blood cell size also known as anisocytosis, is an emerging biomarker of chronic disease morbidity and mortality [1]. RDW is associated with increased risk of cardiovascular diseases (CVD) including coronary artery disease, heart failure, atherosclerosis, and venous thromboembolism independent of traditional CVD risk factors [2–7]. Elevated RDW was also associated with overall and disease-specific mortality risk [6, 8–11]. Most of these studies, however, were conducted in older individuals, and hence the role of RDW on mortality risk in younger individuals is poorly understood. Although the specific biological mechanisms behind the association between RDW and adverse health outcomes have not been fully explained, it was suggested that it could be mediated through inflammatory cytokines, or oxidative stress [2, 9, 12]. RDW is influenced by factors such as sex, race, body mass index (BMI), cigarette smoking, markers of systemic inflammation and lipids although results were inconclusive [6, 7, 12, 13]. Whereas genetic variants in loci including *G6PD*, *CD36*, and *NOL4L* have been linked with RDW level [14, 15], the interplay between environmental factors and genetic variants on RDW has not yet been investigated.

While the age-adjusted mortality rate in the United States has been declining, there remains a gap in premature mortality and life expectancy among racial/ethnic groups and individuals of various socioeconomic status [16, 17]. Besides, Case and Deaton recently reported that in the past 10–15 years there has been an alarmingly increasing mortality rate among white American men and women aged 30–54 years [18]. Established risk factors of mortality include tobacco use, obesity, and clinical markers such as elevated blood pressure and hyperlipidemia [19]. The growing mortality disparity and widening of life expectancy gap suggest a need to identify additional biomarkers to improve early detection of at-risk individuals to enhance precise and targeted interventions.

This new evidence of sharply declining longevity not only among African Americans (AA) but also young whites has made us reevaluate the role of RDW in younger populations of urban AA and whites since RDW was shown to be a good predictor of mortality for those in middle and old age [11]. Using prospectively collected data (August 2004–December 2013) from the Healthy Aging in Neighborhoods of Diversity across the Life Span (HANDLS) study, which is a large longitudinal study of socioeconomically diverse community-dwelling AA and white working-age adults, the objectives of this study were (1) to assess the association between RDW and all-cause and cause-specific mortality; (2) to identify lifestyle and environmental determinants of RDW; and (3) to explore the effect of gene × environment interaction on RDW.

## Methods

### Population and study design

The HANDLS study is a large population-based prospective longitudinal study conducted in Baltimore, MD. Study design and methods of data collection have been described previously [20]. Briefly, the HANDLS study was designed to establish a single-site study for the investigation of age-related health disparities in socioeconomically diverse adult AAs and whites. The initial wave of data collection recruited 3720 individuals from 2004 to 2009. Participants were men and women aged 30–64 years, above and below 125% of the 2004 US Federal poverty level. At baseline, 2744 participants had complete RDW data. We excluded 18 subjects with sickle cell disease leaving 2726 for the current analysis.

### Red cell distribution width and covariates

During the baseline data collection (2004–2009), study participants were interviewed using structured questionnaires, underwent medical examinations, and provided social and medical histories as well as a blood sample. Data included age, sex, race, poverty status, BMI (kg/m^2^), waist-hip ratio (WHR), current smoking status, current alcohol use, highest education level attained and reported illicit drug use (e.g., marijuana, cocaine). RDW was measured by automated Coulter DXH 800 hematology analyzer as part of peripheral complete blood count (Beckman Coulter, Brea, CA), and was expressed as coefficient of variation (%) of red blood cell volume distribution. Regular calibration was performed every 3 months on the hematology analyzer and quality control was performed according to the manufacturer’s recommendations. Hypertension was defined as self-reported history of hypertension, use of anti-hypertensive medications, or blood pressure of > 140/90. Diabetes mellitus was defined as self-reported history of Type I or II diabetes mellitus, reported use of diabetes medication, or fasting plasma glucose ≥ 126 mg/dl. Self-reported depressive symptoms were assessed using the Center for Epidemiologic Studies-Depression (CES-D) scale [21]. Additional covariates included hemoglobin, serum iron, serum vitamin B12, total white blood cell (WBC) count, erythrocyte sedimentation rate (ESR), high-sensitivity C-reactive protein (hsCRP), low density lipoprotein-cholesterol (LDL), and estimated glomerular filtration rate (eGFR) [22].

### Mortality and cause of death assessment

Mortality was ascertained through the National Death Index (NDI) database. Each study participant was matched to the NDI using name, date and state of birth, sex, race, maiden name and social security number [16, 23]. Participants were followed from the date of study enrolment to date of death or censored date, December 31, 2013. Primary cause of death is based on the International Classification of Diseases 10th edition (ICD-10). Death due to cardiovascular diseases included the ICD codes from I10.0 to I82.9. A total of 2704 participants had complete follow-up and mortality outcome data.

### Genotyping

A subset of AA participants was genotyped using Illumina 1 M and Illumina 1 M-Duo genotyping arrays (Illumina, San Diego, CA). Genotype calling and quality control followed standard procedures. Samples with call rate < 95%, sex mismatch, ethnic ancestry outliers and cryptic related individuals were excluded. SNPs with minor allele frequency less than 1%, Hardy–Weinberg equilibrium *p* value of less than 1.0 × 10^−7^, call rate < 95% were excluded. After sample and genotype quality control, 1024 AAs had complete genome-wide genotype data. Genotype data management and quality control was performed using PLINK (https://www.cog-genomics.org/plink2) [24]. Principal components were generated using a set of linkage disequilibrium based pruned independent single nucleotide polymorphisms. This set of single nucleotide polymorphisms were selected under PLINK default settings of pairwise correlation coefficient of 0.2 between single nucleotide polymorphisms in a sliding window of size of 50. The correlation coefficient of 0.2 was used to select near completely independent SNPs to avoid collinearity between them. To increase power, genotypes were imputed using the 1000 Genomes Project phase 1 version 3 reference panel. Genotypes were phased using the MaCH software and imputation was performed using minimac software (http://genome.sph.umich.edu/wiki/MaCH).

### Statistical analysis

Distributions of continuous variables were plotted using histograms and were checked for skewness. To achieve normal distribution, hsCRP, ESR, and WBC count were natural-logarithm transformed. Categorical variables included in the analysis were: sex, race (AA/white), poverty status (above/below), college degree (yes/no), current cigarette smoking (yes/no), current alcohol use (yes/no), hypertension (yes/no), diabetes mellitus (yes/no), CES-D score (< 16/≥ 16), marijuana use (yes/no), current cocaine use (yes/no).

### Survival analysis

We fitted unadjusted and multivariable adjusted Cox proportional hazards regression models to estimate hazard ratios and 95% confidence intervals (CI) of all-cause mortality and CVD-specific mortality. Age at study entrance and exit (death or censored date) were used as the measurement of follow-up time [25]. To allow a balanced comparison of mortality risk across the different strata, RDW was categorized by quartiles (cut-offs: 13.2, 13.8, and 14.6%). We initially adjusted for age, sex, race, and poverty status. The full Cox regression model was also adjusted for established predictors of mortality and potential confounders: current cigarette smoking, BMI, LDL, hypertension and diabetes mellitus. Test for linear trend across the categories of RDW was performed by assigning the median value to each quartile of RDW and included this variable as a continuous term in the full Cox regression model. The proportional hazards assumption was checked by inspection of log–log survival curves, and formally evaluated using the scaled Schoenfeld residuals method yielding p-values > 0.1 suggesting the proportional hazards assumptions were not violated. Although for the sake of parsimony we adjusted the full Cox regression model for the above nine variables, we performed sensitivity analyses by adding the following variables one at time to the full model, and simultaneously adjusting for them: WHR, systolic blood pressure, hemoglobin, serum iron, serum vitamin B12, ESR, hsCRP, WBC count, and eGFR.

To assess the association between sickle cell trait (SCT) and RDW and its role on the association between RDW and mortality risk, we used *HBB*-rs334 (*A* > *T*, p.Glu6Val, minor allele frequency = 6.2%) to determine SCT status among study participants. We compared RDW level between non-carriers and carriers of the sickle cell allele. Main effects of SCT as well as interaction between RDW and SCT on all-cause and CVD-specific mortality risk were estimated.

We explored effect modifications by established predictors of mortality by including a multiplicative interaction term in the full Cox regression model. The variables tested for interaction were age, sex, race, poverty status, current cigarette smoking, BMI, LDL, hypertension, and diabetes mellitus. Evidence of interaction was tested by comparing the models with and without the interaction term using a likelihood ratio test.

To assess the performance of RDW as predictive marker of all-cause and CVD-specific mortality we assessed model calibration of the fully adjusted Cox regression models using the modified Nam-D’Agostino goodness-of-fit (GOF) test for survival data [26].

### Determinants of red cell distribution width

To identify determinants of RDW, we fitted multiple linear regression models to estimate beta coefficients and 95% CIs. The initial model included age, sex, race, and poverty status; and the full model was further adjusted for potential confounders and previously reported determinants of RDW [5, 6] including smoking status, BMI, hypertension, diabetes mellitus, LDL, eGFR, and hsCRP. Sensitivity analyses were performed by further adjusting the full model for hemoglobin levels.

### Gene × environment interaction analysis in RDW

Among AAs (N = 998) who had complete RDW and genotype data, gene × environment interactions were assessed between lifestyle factors and genetic variants by including an interaction term into the regression model adjusted for age, sex and the first five principal components to account for population stratifications. The variants tested were: *TMEM57*-*RHD*-rs10903129, *NOL4L*-rs4911241, *CD36*-rs3211938, *LINC01184*-*SLC12A2*-rs10063647, *LINC01184*-*SLC12A2*-rs10089, *TRIB1*-rs2954029 [14, 15]. These variants were selected since they were identified by one of the largest genetic association studies and may have biological significance in red cell biology. All statistical tests were two-sided, and p-value < 0.05 was considered significant. Data analyses were performed using the R statistical software version 3.2.3 (https://www.R-project.org/) [27].

## Results

The characteristics of the 2726 study participants in the present analyses stratified by race are shown in Table 1: 57% were AAs, 56% were women, and baseline mean age (standard deviation) was 48.1 (9.2) years. The mean (standard deviation) of RDW was 14.1% (1.7).

**Table 1:** Participant characteristics and mortality outcomes stratified by race

Characteristics	African Americans		Whites	
	N = 1563	%	N = 1163	%
Age, mean (SD)	48.0 (9.2)		48.2 (9.3)	
Sex				
Women	880	56.3	658	56.6
Men	683	43.7	505	43.4
Poverty status				
Above	823	52.7	788	67.8
Below	740	47.3	375	32.2
Education-college degree				
No	1458	93.8	913	82.9
Yes	97	6.2	188	17.1
Missing	8		62	
Current smoking status				
No	686	49.9	574	54.4
Yes	688	50.1	481	45.6
Missing	189		108	
Current alcohol use				
No	597	43.6	426	40.3
Yes	773	56.4	630	59.7
Missing	193		107	
Marijuana use				
No	1141	83.3	952	90.2
Yes	228	16.7	104	9.8
Missing	194		107	
Current cocaine use				
No	1271	92.4	1016	95.8
Yes	104	7.6	45	4.2
Missing	188		102	
CES-D scale score				
< 16	859	58.9	661	59
≥ 16	600	41.1	460	41
Missing	104		42	
Hypertension				
No	712	48.6	660	58.9
Yes	752	51.4	461	41.1
Missing	99		42	
Diabetes mellitus				
No	1209	81.9	937	83.3
Yes	268	18.1	188	16.7
Missing	86		38	
RDW (%)	14.48 (1.91)		13.80 (1.32)	
BMI (kg/m^2^)	29.95 (7.97)		30.21 (7.77)	
LDL (mg/dL)	107.16 (37.13)		111.94 (35.98)	
eGFR (ml/min/1.73 m^2^)	102.51 (22.72)		96.14 (17.72)	
Total WBC (10^9^/L)	6.18 (2.04)		7.04 (2.28)	
hsCRP (mg/L)	5.47 (12.10)		4.45 (6.62)	
WHR	0.91 (0.08)		0.95 (0.39)	
All-cause mortality	141		85	
CVD-specific mortality	48		24	

*BMI* body mass index, *CES-D* Center for Epidemiologic Studies Depression Scale, *hsCRP* high-sensitivity C-reactive protein, *CVD* cardiovascular disease, *eGFR* estimated glomerular filtration rate, *LDL* low density lipoprotein cholesterol, *RDW* red cell distribution width, *SD* standard deviation, *WBC* white blood cell count, *WHR* waist-hip ratio

### Association of RDW with all-cause mortality

Participants were followed for up to 9.4 years (median follow-up time = 6.8 years). After 18,424 person-years of follow-up time, there were a total of 226 deaths. CVD was the leading cause death in our cohort [72/226 (31.9%)] (Table 1). The unadjusted hazard ratio (HR) for all-cause mortality among participants in the highest quartile of RDW was 1.90 (95% CI 1.30–2.78, p-trend < 0.001) compared to those in the lowest quartile. After adjusting for age, sex, race and poverty status, the risk of all-cause mortality for people in the highest RDW quartile was 1.95 (95% CI 1.32–2.88, p-trend < 0.001). In the full model adjusted for age, sex, race, poverty status, smoking status, BMI, LDL, hypertension and diabetes mellitus, participants in the highest quartile had a 73% increased risk of all-cause mortality (95% CI 1.10–2.74, p-trend = 0.006) (Table 2). To identify subgroup effects of RDW on all-cause mortality risk, we performed stratified analyses. The results of stratified analysis by sex, race, poverty status, current smoking and BMI for all-cause mortality are presented in Table 3. We observed a statistically significant interaction between RDW and BMI (p-interaction = 0.004). The increased risk of death associated with higher RDW level was stronger among individuals with BMI ≥ 25.0 (adjusted HR for the highest quartile vs. lowest quartile = 2.06, 95% CI 1.11–3.83, p-trend = 0.004). There was no effect modification by the other variables considered (p-interaction > 0.05). Results from the sensitivity analyses after controlling for additional covariates including hemoglobin, serum iron and serum vitamin B_12_ were consistent with the main results from the full Cox proportional hazards regression model.

**Table 2:** Risk of all-cause and cardiovascular disease-specific mortality and red cell distribution width

RDW^a^	Total	Events	HR^b^ (95% CI)	HR^c^ (95% CI)	HR^d^ (95% CI)
All-cause mortality					
RDW-Q1	638	41	1.00	1.00	1.00
RDW-Q2	743	56	1.19 (0.79–1.78)	1.17 (0.78–1.76)	1.06 (0.66–1.72)
RDW-Q3	647	50	1.12 (0.74–1.7)	1.13 (0.74–1.71)	1.08 (0.67–1.75)
RDW-Q4	676	79	1.90 (1.30–2.78)	1.95 (1.32–2.88)	1.73 (1.10–2.74)
P-trend^e^			< 0.001	< 0.001	0.006
CVD-specific mortality					
RDW-Q1	605	8	1.00	1.00	1.00
RDW-Q2	702	15	1.60 (0.68–3.78)	1.55 (0.65–3.67)	0.92 (0.34–2.49)
RDW-Q3	613	16	1.79 (0.77–4.18)	1.73 (0.73–4.09)	1.09 (0.41–2.85)
RDW-Q4	630	33	4.03 (1.86–8.74)	3.89 (1.75–8.62)	2.49 (1.03–6.05)
P-trend^e^			< 0.001	< 0.001	0.004

*CI* confidence interval, *CVD* cardiovascular diseases, *HR* hazard ratio

^a^RDW, red-cell distribution level in quartiles. Quartile cut-off points were 13.2, 13.8, and 14.6%

^b^Unadjusted Cox proportional hazards regression model

^c^Adjusted for age, sex, race, and poverty status

^d^Adjusted for age, sex, race, poverty status, smoking status, body mass index, low density lipoprotein cholesterol, diagnosis of hypertension, and diagnosis of diabetes mellitus

^e^Linear trend test was performed using median of the quartile as a continuous variable in the Cox proportional hazards regression model

**Table 3:** Stratified analysis of the association between red cell distribution width and all-cause mortality

RDW^a^	HR^b^ (95% CI)	HR^b^ (95% CI)	P-interaction^c^
Sex	Men	Women	
RDW-Q1	1.00	1.00	0.42
RDW-Q2	1.39 (0.73–2.67)	0.71 (0.34–1.47)	
RDW-Q3	1.20 (0.60–2.38)	0.85 (0.42–1.7)	
RDW-Q4	2.41 (1.26–4.59)	1.17 (0.6–2.28)	
P-trend^d^	0.006	0.29	
Race	African Americans	Whites	
RDW-Q1	1.00	1.00	0.35
RDW-Q2	0.76 (0.4–1.44)	1.68 (0.79–3.59)	
RDW-Q3	0.86 (0.46–1.58)	1.57 (0.71–3.49)	
RDW-Q4	1.18 (0.66–2.1)	3.60 (1.68–7.72)	
P-trend^d^	0.22	0.001	
Poverty status	Above	Below	
RDW-Q1	1.00	1.00	0.17
RDW-Q2	1.62 (0.76–3.49)	0.85 (0.45–1.61)	
RDW-Q3	2.17 (1.03–4.56)	0.63 (0.32–1.24)	
RDW-Q4	2.80 (1.33–5.92)	1.33 (0.74–2.4)	
P-trend^d^	0.005	0.18	
Current smoking	No	Yes	
RDW-Q1	1.00	1.00	0.42
RDW-Q2	1.31 (0.61–2.81)	1.04 (0.56–1.94)	
RDW-Q3	0.82 (0.34–1.99)	1.28 (0.71–2.31)	
RDW-Q4	1.83 (0.82–4.06)	1.82 (1.03–3.23)	
P-trend^d^	0.17	0.01	
BMI	BMI < 25.0	BMI ≥ 25.0	
RDW-Q1	1.00	1.00	0.004
RDW-Q2	1.16 (0.59–2.28)	0.89 (0.44–1.8)	
RDW-Q3	0.79 (0.37–1.69)	1.34 (0.7–2.55)	
RDW-Q4	1.44 (0.7–2.95)	2.06 (1.11–3.83)	
P-trend^d^	0.39	0.002	

*BMI* body mass index, *CI* confidence intervals, *HR* hazard ratio

^a^RDW, red cell distribution width levels in quartiles. Quartile cut-off points were 13.2, 13.8, and 14.6%

^b^Multivariable adjusted Cox proportional hazards regression model

^c^p-value from likelihood ratio test of interaction

^d^Linear trend test was performed using median of the quartile as a continuous variable in the Cox proportional hazards regression model

### Association of RDW with CVD-specific mortality

Similarly, higher RDW level was associated with an increased risk of CVD-specific mortality. In the multivariable adjusted model, HR for the highest quartile was 2.49 (95% CI 1.03–6.05, p-trend = 0.004) (Table 2). The increased risk of CVD-specific mortality associated with higher RDW was more pronounced among participants with BMI ≥ 25 (p-interaction = 0.05) (Additional file 1: Table S1).

SCT was not associated with all-cause and CVD-specific mortality. There was also no effect modification or confounding by SCT on the association between RDW and increased mortality risk. Similarly, RDW levels did not differ between non-carriers and carriers of the sickle cell allele (data not shown). Risk estimates of BMI and LDL on all-cause mortality and CVD-specific mortality are shown in Additional file 1: Table S2.

Results of model calibration of RDW in the fully adjusted Cox regression model was *P*_*GOF*_ = 0.445 (χ^2^ = 3.718, df = 4) for all-cause mortality, and *P*_*GOF*_ = 0.965 (χ^2^ = 0.071, df = 2) for CVD-specific mortality indicating that the models were not miscalibrated (Additional file 1: Figure S1).

### Determinants of RDW

#### Replication of previously identified RDW determinants

We replicated known predictors of RDW (Additional file 1: Table S3). In the full multivariable adjusted linear regression model, RDW was significantly higher among AAs compared to whites [beta (b) (95% CI) = 0.6 (0.5, 0.8)], and current smokers compared to non-current smokers [0.3 (0.1, 0.4)], while RDW was lower among men compared to women [− 0.3 (− 0.5, − 0.2)]. There was a direct association between RDW and BMI, and hsCRP. On the other hand, LDL [− 0.005 (− 0.007, − 0.003)], and eGFR [− 0.005 (− 0.01, − 0.001)] showed an inverse association with RDW (Additional file 1: Table S3).

#### Novel determinants of RDW

We identified a novel significant association between RDW and illicit drug use: marijuana use [yes vs. no; − 0.3 (− 0.5, − 0.03)], current cocaine use [yes vs. no; − 0.4 (− 0.7, − 0.02)]. WHR was positively associated with RDW [1.4 (0.2, 2.5)] (Table 4). Stratified analyses by sex, poverty status, and race showed that both marijuana [b (95% CI) = − 0.6 (− 1.0, − 0.2)], and cocaine use [b (95% CI) = − 0.6 (− 1.2, − 0.1)] were associated with RDW among women, and the effect of current cocaine use was limited to participants below poverty level [b (95% CI) = − 0.6 (− 1.1, − 0.2)] (Table 4).

**Table 4:** Association between red cell distribution width and waist-hip ratio, and illicit drug use

Characteristics	Beta^a^	95% CI	Beta^b^	95% CI
Overall population				
WHR	0.3	0.02, 0.5	1.4	0.2, 2.5
Marijuana use				
No	Ref		Ref	
Yes	− 0.2	− 0.4, − 0.01	− 0.3	− 0.5, − 0.03
Current cocaine use				
No	Ref		Ref	
Yes	− 0.3	− 0.6, − 0.02	− 0.4	− 0.7, − 0.02
Stratified analysis by sex				
Men				
WHR	1.0	− 0.1, 2	0.3	− 1.4, 2.1
Marijuana use				
No	Ref		Ref	
Yes	− 0.04	− 0.2, 0.2	− 0.002	− 0.3, 0.3
Current cocaine use				
No	Ref		Ref	
Yes	− 0.02	− 0.3, 0.3	− 0.1	− 0.5, 0.3
Women				
WHR	0.2	− 0.1, 0.5	1.6	0.1, 3.2
Marijuana use				
No	Ref		Ref	
Yes	− 0.3	− 0.7, − 0.01	− 0.6	− 1.0, − 0.2
Current cocaine use				
No	Ref		Ref	
Yes	− 0.6	− 1.1, − 0.1	− 0.6	− 1.2, − 0.1
Stratified analysis by race				
African American				
WHR	2.5	1.2, 3.7	1.6	− 0.1, 3.4
Marijuana use				
No	Ref		Ref	
Yes	− 0.2	− 0.5, 0.03	− 0.3	− 0.6, 0.03
Current cocaine use				
No	Ref		Ref	
Yes	− 0.3	− 0.7, 0.1	− 0.3	− 0.8, 0.1
White				
WHR	0.1	− 0.1, 0.3	0.6	− 0.7, 2.0
Marijuana use				
No	Ref		Ref	
Yes	− 0.1	− 0.4, 0.2	− 0.2	− 0.5, 0.2
Current cocaine use				
No	Ref		Ref	
Yes	− 0.3	− 0.6, 0.1	− 0.4	− 0.9, 0.1
Stratified analysis by poverty status				
Below poverty				
WHR	1.8	0.4, 3.2	0.3	− 1.5, 2.1
Marijuana use				
No	Ref		Ref	
Yes	− 0.3	− 0.6, 0.1	− 0.3	− 0.7, 0.1
Current cocaine use				
No	Ref		Ref	
Yes	− 0.5	− 0.9, − 0.1	− 0.6	− 1.1, − 0.1
Above poverty				
WHR	0.2	− 0.03, 0.4	2.1	0.7, 3.6
Marijuana use				
No	Ref		Ref	
Yes	− 0.1	− 0.4, 0.1	− 0.3	− 0.6, 0.05
Current cocaine use				
No	Ref		Ref	
Yes	0.01	− 0.4, 0.4	0.03	− 0.5, 0.5

*CI* confidence interval, *WHR* waist-hip ratio

^a^Adjusted for age, sex, race, and poverty status

^b^Full model adjusted for age, sex, race, poverty status, current cigarette smoking, body mass index, diagnosis of hypertension, diagnosis of diabetes mellitus, low density lipoprotein cholesterol, estimated glomerular filtration rate, and high-sensitivity C-reactive protein

#### Gene × environment interaction in RDW

After correcting for multiple testing using the Bonferroni method, there was no evidence of gene × environment interaction between RDW linked genetic variants (*TMEM57*-*RHD*-rs10903129, *NOL4L*-rs4911241, *CD36*-rs3211938, *LINC01184*-*SLC12A2*-rs10063647, *LINC01184*-*SLC12A2*-rs10089, *TRIB1*-rs2954029) and non-genetic factors previously known to influence RDW (Additional file 1: Table S4).

## Discussion

In the present study, using prospectively collected clinical, genetic and mortality data, we assessed the association between RDW and all-cause and disease-specific mortality risk, as well as investigated determinants of RDW. Elevated RDW conferred a 1.73-times increased risk of all-cause mortality and a 2.49-times increased risk of CVD-specific mortality in urban adults. This elevated risk was substantially modified by BMI. Our results confirmed previously reported predictors of RDW such as cigarette smoking, BMI and CRP, and identified novel associations between RDW and illicit drug use: marijuana and current cocaine use, and WHR.

Our findings of positive associations between RDW and premature mortality among younger urban adults are consistent with previous reports of elevated RDW and all-cause and CVD-specific mortality among middle and older age individuals. Two separate studies using the National Health and Nutrition Examination Survey (NHANES) (1988–1994) data reported that the highest quantile of RDW was associated with a 2-fold, and 2.1- to 2.3-fold increased risk of all-cause and CVD-specific mortality, respectively [9, 10]. In a meta-analysis of RDW and mortality in older individuals, Patel et al. reported a positive association between RDW and all-cause and cause specific mortalities due to CVD, cancer, and other causes [11]. It should be noted that our cohort represents adults younger (mean age = 48.1 years) than participants included in the NHANES study (mean age = 62.0 years) [9], and the meta-analysis (mean age ranged 73.6–79.1 years) [11]. Also, the presents study contains a large proportion of AAs (57%) compared to 16.9% in previous studies of RDW and mortality [11].

Contrary to the declining mortality rate trends seen in industrialized countries, the recent rise of premature mortality rates among US adults in their prime-age is a serious clinical and public health problem [18]. Most of these early deaths, measured as years of life lost, were due mainly to preventable factors such as ischemic heart disease, drug and alcohol use disorders which are themselves multifactorial in origin [18, 19, 28]. While some of these factors (socioeconomic factors, tobacco use, drug and alcohol use, sedentary lifestyle) have long been the focus of clinical and public health prevention strategies, there is a substantial disparity in premature mortality, particularly among young vulnerable and disadvantaged individuals [16, 17, 29]. To improve targeted and precise interventions and eliminate this disparity gap, identifying readily available and less expensive biomarkers of premature mortality is important. The fact that RDW is simple and routinely measured clinical parameter makes it a useful tool for health prevention intervention strategies.

RDW reflects variations in red blood cell size (anisocytosis). In addition to aiding the clinical evaluation of anemia, RDW is independently associated with chronic disease risk and mortality [6]. The exact pathophysiological mechanisms behind this association are not clearly understood although inflammatory cytokines, oxidative stress, and neurohumoral factors are implicated as potential mediators [2, 9, 12, 30]. Inflammatory cytokines such as interleukin-1, tumor necrosis factor-α, and interferon-γ, which are known to be released in chronic inflammatory states, could affect bone marrow red blood cell production, maturation, and could subsequently lead to anisocytosis [31]. Although we found a positive association between RDW and inflammation markers, it should be noted that the effect of RDW on both all-cause and cardiovascular mortality risk was independent of hsCRP and ESR—two commonly used markers of systemic inflammation. On the other hand, oxidative stress and systemic inflammation have a complex interrelationship and they are associated with morbidity and mortality. A recent study conducted in elderly individuals showed that serum oxidative stress markers (e.g., derivatives of reactive oxygen metabolites and total thiol levels) were significantly associated with all-cause mortality [32]. However, following adjustment for CRP, the effect of derivatives of reactive oxygen metabolites on mortality was no longer significant. Further, the positive correlation between derivatives of reactive oxygen metabolites and CRP suggested the effect of these non-specific markers of oxidative stress might partly be explained by inflammation [32]. To date, there are no studies that directly investigated oxidative stress and RDW in relation to mortality risk and thus we cannot rule out that the effect of increased RDW on mortality is not mediated through oxidative stress processes. To understand the role of SCT on the association between RDW and mortality risk, we assessed the confounding effect of SCT and interaction between SCT and RDW. We found no evidence of a main effect, interaction or confounding effect for SCT. Together with the results of model calibration analyses, these data indicate that RDW could be a valuable predictive biomarker of mortality to identify not only older individuals but also younger adults at higher risk of premature mortality. Future studies utilizing red blood cell specific oxidative stress markers such as fluorescent heme degradation products [33] which are not associated with CRP [34] could shed light on the causal link between RDW, oxidative stress and mortality risk.

The findings in the present study that sex, race, cigarette smoking, BMI, and hsCRP influence RDW are consistent with previous reports [6, 7, 12, 13]. In the present study, we provide the first evidence that WHR and illicit drug use as novel independent determinants of RDW. The positive association between WHR and RDW independent of BMI and other factors known to influence RDW suggests that central obesity as novel predictor of RDW, and could shine light as to how RDW is associated with chronic disease morbidity and mortality.

Interestingly, we found inverse associations between marijuana and cocaine use and RDW independent of potential confounders including systemic inflammation markers. There are no experimental data on the link between RDW, cocaine, and ∆^9^-tetrahydrocannabinol (THC). In humans, little is known about the effect of illicit drug use on red blood cell physiology. Two small studies that assessed the effect of cocaine on bone marrow function reported conflicting findings. A study by Siegel et al. showed cocaine use resulted in erythrocytosis while another study by Weber et al. found no significant association between cocaine use and red blood cell count as well as reticulocyte count suggesting that cocaine use might not have effect on bone marrow mediated erythropoiesis [35, 36]. Cocaine use is known to have negative effects including raised blood pressure and oxidative stress damage to cardiac tissues [37]. Although cannabinoids regulate the production of inflammatory mediators [38] and sometimes prescribed by physicians for therapeutic benefits, recent reports indicate that marijuana (natural or synthetic toxic street form) is associated with production of reactive oxygen species, and cardiovascular diseases [39]. These findings imply that, in the context of the current drug use epidemic, the significance of RDW as a marker of mortality requires additional evaluation. Further experimental studies are required to elucidate the molecular mechanism behind illicit drug use and RDW. We did not find a significant gene x environment interaction in our targeted analysis after correcting for multiple testing. This could be due to the smaller sample size with genotype data. Future interaction studies on larger samples using genome-wide sequence variants are required to understand the influence of environmental exposure on genes that affect RDW levels.

The limitations of the present study include the following: first, the lack of genotype data among white participants that precluded gene × environment interaction analysis. Second, the absence of objectively measured data in our cohort on potential confounders such as serum nutrients and antioxidants, although a previous study showed serum antioxidants did not change the effect of RDW on mortality [9]. Third, the smaller number of subjects with specific causes of death limited further disease-specific mortality analyses.

The strengths of this study are its large sample size, inclusion of participants with diverse race and socioeconomic status, and availability of a wide array of data on epidemiologic, environmental, clinical (e.g., high-sensitivity CRP) and genomic data. Our results should be applicable to similar urban dwelling communities. Compared to the NHANES study which was collected between 1988 and 1994 and included rural and urban communities, our study used recently collected data (2004–2013), and was comprised of younger adults from a single urban study site, minimizing unmeasured confounding by geographic localities. Previous studies of RDW were conducted mostly in the elderly, and individuals of European ancestry [11]. By focusing on racially diverse younger individuals who are at risk of health disparity, we showed that the adverse effects of elevated RDW could be seen earlier in the life course.

## Conclusions

In summary, RDW is strongly associated with mortality in urban AA and white adults that was modified by obesity. Our findings have several implications: first, RDW is an attractive target for intervention strategies as it is simple and readily available. Second, RDW could be used to identify vulnerable population groups with substantial burdens of premature death for precise and tailored interventions on modifiable factors of mortality. Clinical and public health prevention strategies could benefit from incorporating emerging biomarkers of mortality such as RDW. Third, during physician office visits, it provides an excellent opportunity for health care providers for individualized assessment, and to look out for preventable and/or treatable causes of premature mortality in subjects with elevated RDW. Because RDW is already included in complete blood count, it would not incur any additional cost for testing unlike other serum biomarkers of disease risk. Our results strengthen the call to broaden the use of RDW beyond its conventional use in the management of anemia [1, 6]. Further research is required to extend our findings, identify best approaches to target RDW, and evaluate its utility in public health surveillance using randomized controlled trials.

## Additional file

**Additional file 1.** Additional Figure S1 and Tables S1–S4.

## Abbreviations

AA: African American
BMI: body mass index
CES-D: Center for Epidemiologic Studies-Depression scale
CI: confidence interval
CVD: cardiovascular disease
ESR: erythrocyte sedimentation rate
eGFR: estimated glomerular filtration rate
HANDLS: Healthy Aging in Neighborhoods of Diversity across the Life Span study
HR: hazard ratio
hsCRP: high-sensitivity C-reactive protein
ICD: International Classification of Diseases
LDL: low density lipoprotein cholesterol
NDI: National Death Index database
RDW: red cell distribution width
WBC: white blood cell count
WHR: waist-hip ratio
