# Supplementary material for: Association of red cell distribution width with all-cause and cardiovascular-specific mortality in African American and white adults: a prospective cohort study
Source: J Transl Med. 2017 Oct 13;15:208. doi: 10.1186/s12967-017-1313-6 (PMC5640961; doi:10.1186/s12967-017-1313-6)
Supplement: Supplementary file 1 — Additional file 1. Additional Figure S1 and Tables S1–S4. [file 12967_2017_1313_MOESM1_ESM.docx]

**Additional Information**

**Association of red cell distribution width with all-cause and cardiovascular-specific mortality in African American and white adults: a prospective cohort study**

Salman M. Tajuddin^1^, e-mail: salman.tajuddin@nih.gov

Mike A. Nalls^2,3^, e-mail: nallsm@mail.nih.gov

Alan B. Zonderman^1^, e-mail: zondermana@mail.nih.gov

Michele K. Evans^1,*^, e-mail: EvansM@grc.nia.nih.gov

^1^ Laboratory of Epidemiology and Population Sciences, National Institute on Aging, National Institutes of Health, Baltimore, MD 21224, USA.

^2^ Laboratory of Neurogenetics, National Institute on Aging, National Institutes of Health, Bethesda, MD, 20892, USA.

^3^ Data Tecnica International LLC, Glen Echo, MD 20812, USA.

*Correspondence: [EvansM@grc.nia.nih.gov](mailto:EvansM@grc.nia.nih.gov)

^1^Laboratory of Epidemiology and Population Sciences

National Institute on Aging, National Institutes of Health

Room # 04C222, Suite 100

251 Bayview Boulevard

Baltimore, MD 21224, USA

Additional Figure S1 Model calibration test using the modified Nam-D’Agostino goodness-of-fit test with observed and expected mortality counts in the HANDLS study. (A) all-cause mortality, (B) cardiovascular-specific mortality.

(A)
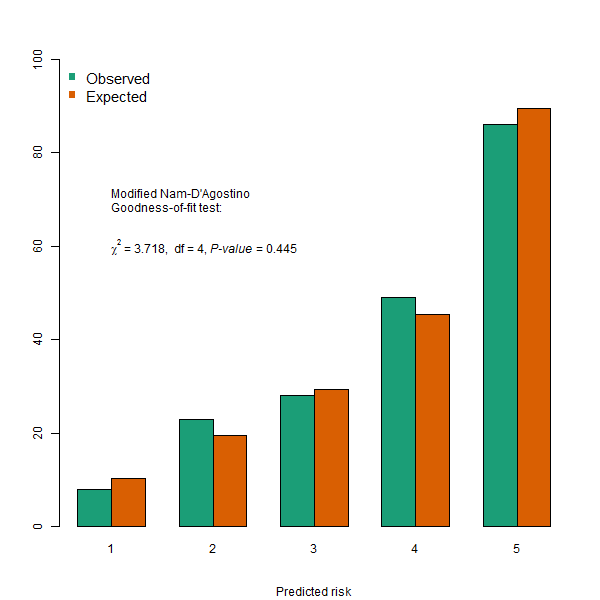


(B)
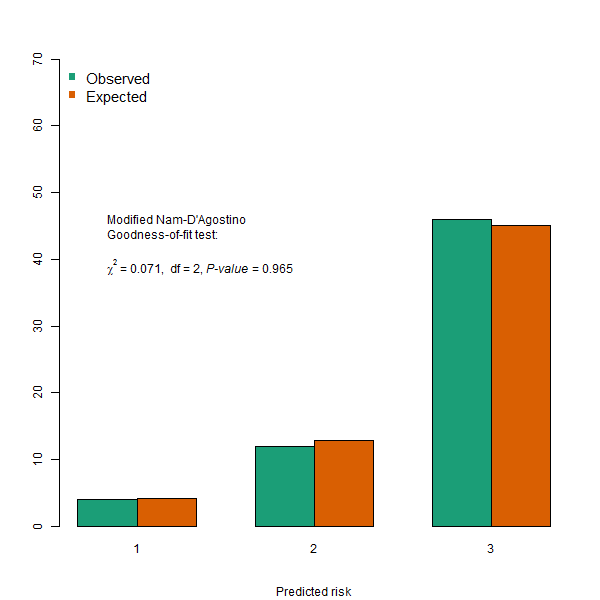


Additional Table S1 Stratified analysis of the association between red cell distribution width and cardiovascular disease-specific mortality

| RDW^a^ | HR^b^ (95% CI) | HR^b^ (95% CI) | P-interaction^c^ |
| --- | --- | --- | --- |
| **Sex** | Men | Women |  |
| RDW-Q1 | 1.00 | 1.00 | 0.07 |
| RDW-Q2 | 2.42 (0.62 - 9.46) | 0.14 (0.02 - 1.27) |  |
| RDW-Q3 | 1.04 (0.22 - 4.83) | 0.91 (0.25 - 3.28) |  |
| RDW-Q4 | 4.93 (1.23 - 19.81) | 1.51 (0.45 - 5) |  |
| P-trend^d^ | 0.02 | 0.05 |  |
| **Race** | African Americans | Whites |  |
| RDW-Q1 | 1.00 | 1.00 | 0.83 |
| RDW-Q2 | 0.66 (0.17 - 2.49) | 1.32 (0.29 - 6.01) |  |
| RDW-Q3 | 0.99 (0.29 - 3.37) | 1.14 (0.23 - 5.71) |  |
| RDW-Q4 | 1.88 (0.61 - 5.81) | 3.64 (0.89 - 14.92) |  |
| P-trend^d^ | 0.04 | 0.05 |  |
| **Poverty status** | Above | Below |  |
| RDW-Q1 | 1.00 | 1.00 | 0.09 |
| RDW-Q2 | 3.86 (0.44 - 33.62) | 0.47 (0.13 - 1.77) |  |
| RDW-Q3 | 5.35 (0.64 - 44.64) | 0.44 (0.12 - 1.61) |  |
| RDW-Q4 | 11.76 (1.48 - 93.44) | 1.18 (0.39 - 3.62) |  |
| P-trend^d^ | 0.002 | 0.37 |  |
| **Current smoking** | No | Yes |  |
| RDW-Q1 | 1.00 | 1.00 | 0.8 |
| RDW-Q2 | 0.73 (0.17 - 3.02) | 1.19 (0.28 - 5.12) |  |
| RDW-Q3 | 0.77 (0.18 - 3.22) | 1.73 (0.44 - 6.84) |  |
| RDW-Q4 | 1.63 (0.45 - 5.97) | 3.92 (1.06 - 14.54) |  |
| P-trend^d^ | 0.23 | 0.006 |  |
| **Obesity** | BMI <25.0 | BMI ≥25.0 |  |
| RDW-Q1 | 1.00 | 1.00 | 0.05 |
| RDW-Q2 | 1.65 (0.3 - 9.15) | 0.59 (0.16 - 2.23) |  |
| RDW-Q3 | 0.41 (0.03 - 4.87) | 1.38 (0.46 - 4.14) |  |
| RDW-Q4 | 2.53 (0.42 - 15.21) | 2.76 (0.98 - 7.78) |  |
| P-trend^d^ | 0.32 | 0.003 |  |
| *BMI*, body mass index; *CI*, confidence interval. | | | |
| ^a^RDW, red cell distribution width levels in quartiles. Quartile cut-off points were 13.2%, 13.8%, and 14.6%. | | | |
| ^b^Multivariable adjusted Cox proportional hazards regression model. | | | |
| ^c^P-value from likelihood ratio test of interaction. | | | |
| ^d^Linear trend test was performed using medians of each quartile as a continuous variable in the Cox proportional hazards regression model. | | | |

Additional Table S2 Risk estimates of body mass index and low-density lipoprotein cholesterol on all-cause and cardiovascular-specific mortality rates

|  | HR^a^ (95% CI) | P-value |
| --- | --- | --- |
| **All-cause mortality** |  |  |
| BMI (< 25) | 1.00 |  |
| BMI (≥25) | 0.56 (0.40 - 0.78) | 0.0007 |
|  |  |  |
| **CVD-specific mortality** |  |  |
| BMI (< 25) | 1.00 |  |
| BMI (≥25) | 0.83 (0.41 - 1.66) | 0.60 |
|  |  |  |
| **All-cause mortality** |  |  |
| LDL-Normal (<129 mg/dL) | 1.00 |  |
| LDL-Bordeline High (130-159 mg/dL) | 0.81 (0.52 - 1.24) | 0.32 |
| LDL-High (≥160 mg/dL) | 0.95 (0.57 - 1.58) | 0.84 |
|  |  |  |
| **CVD-specific mortality** |  |  |
| LDL-Normal (<129 mg/dL) | 1.00 |  |
| LDL-Bordeline High (130-159 mg/dL) | 0.80 (0.36 - 1.82) | 0.60 |
| LDL-High (≥160 mg/dL) | 1.62 (0.77 - 3.42) | 0.21 |
| *BMI,* body mass index; *CI*, confidence interval; *CVD*, cardiovascular diseases; *HR*, hazard ratio; *LDL,* low-density lipoprotein cholesterol. | | |
| ^a^Multivariable adjusted Cox proportional hazards model. | | |

Additional Table S3 Previously reported predictors of red cell distribution width

| Characteristics | Beta^a^ | 95% CI |
| --- | --- | --- |
| Age | -0.007 | -0.02, 0.003 |
| Sex |  |  |
| Women | Ref |  |
| Men | -0.3 | -0.5, -0.2 |
| Race |  |  |
| White | Ref |  |
| African American | 0.6 | 0.5, 0.8 |
| Poverty status |  |  |
| Above | Ref |  |
| Below | 0.0001 | -0.2, 0.2 |
| Education-college degree |  |  |
| No | Ref |  |
| Yes | -0.3 | -0.6, -0.08 |
| Current cigarette smoking |  |  |
| No | Ref |  |
| Yes | 0.3 | 0.1, 0.4 |
| Current alcohol use |  |  |
| No | Ref |  |
| Yes | -0.1 | -0.3, 0.05 |
| CESD score |  |  |
| < 16 | Ref |  |
| ≥ 16 | 0.006 | -0.2, 0.2 |
| BMI (kg/m2) | 0.02 | 0.006, 0.03 |
| Total WBC (log) (109/L) | -0.1 | -0.4, 0.1 |
| LDL (mg/dL) | -0.005 | -0.007, -0.003 |
| eGFR (ml/min/1.73 m2) | -0.005 | -0.01, -0.001 |
| hsCRP (log) (mg/L) | 0.1 | 0.04, 0.2 |
| ESR (log) (mm/hr) | 0.07 | -0.02, 0.2 |
| *BMI*, body mass index; *CES-D*, Center for Epidemiologic Studies Depression Scale; *CI*, confidence interval; *hsCRP*, high-sensitivity C-reactive protein; *eGFR*, estimated glomerular filtration rate; *ESR*, erythrocyte sedimentation rate; *LDL*, low density lipoprotein cholesterol; *RDW*, red cell distribution width; *WBC*, white blood cell count; *WHR*, waist-hip ratio. | | |
| ^a^Fully adjusted multiple linear regression model. | | |

Additional Table S4. Gene-environment interaction on red cell distribution width. Significant results at nominal p-value are shown in boldface.

| Variants | Beta^a^ | 95% CI | P-interaction |
| --- | --- | --- | --- |
| rs10903129 (*TMEM57-RHD*, intron) |  |  |  |
| Current cigarette smoking | -0.283 | -0.678, 0.113 | 0.16 |
| BMI | -0.013 | -0.037, 0.012 | 0.30 |
| LDL | 0.001 | -0.005, 0.006 | 0.75 |
| **eGFR** | **0.012** | **0.003, 0.022** | **0.01** |
| hsCRP (log) | -0.095 | -0.222, 0.031 | 0.14 |
| Education | 0.087 | -0.703, 0.877 | 0.83 |
| Marijuana use | 0.166 | -0.363, 0.694 | 0.54 |
| Current cocaine use | -0.04 | -0.91, 0.829 | 0.93 |
| WHR | 0.233 | -2.3, 2.767 | 0.86 |
| rs10063647 (*LINC01184- SLC12A2*, intron) |  |  |  |
| Current cigarette smoking | -0.023 | -0.434, 0.387 | 0.91 |
| BMI | -0.004 | -0.031, 0.023 | 0.78 |
| LDL | -0.003 | -0.008, 0.003 | 0.36 |
| eGFR | -0.007 | -0.018, 0.004 | 0.25 |
| hsCRP (log) | -0.04 | -0.181, 0.101 | 0.58 |
| Education | 0.266 | -0.593, 1.125 | 0.54 |
| Marijuana use | 0.185 | -0.366, 0.737 | 0.51 |
| Current cocaine use | -0.073 | -0.825, 0.68 | 0.85 |
| WHR | 0.384 | -2.535, 3.302 | 0.80 |
| rs10089 (*LINC01184- SLC12A2*, 5'UTR) |  |  |  |
| Current cigarette smoking | -0.017 | -0.5, 0.466 | 0.94 |
| BMI | 0.0004 | -0.026, 0.027 | 0.98 |
| LDL | 0.005 | -0.002, 0.011 | 0.14 |
| eGFR | 0.005 | -0.007, 0.016 | 0.41 |
| hsCRP (log) | -0.02 | -0.182, 0.142 | 0.81 |
| Education | 0.46 | -0.402, 1.323 | 0.30 |
| Marijuana use | -0.129 | -0.807, 0.55 | 0.71 |
| Current cocaine use | -0.636 | -1.573, 0.302 | 0.18 |
| WHR | 2.113 | -1.135, 5.361 | 0.20 |
| rs3211938 (*CD36*, nonsense mutation) |  |  |  |
| **Current cigarette smoking** | **0.579** | **-0.027, 1.184** | **0.06** |
| BMI | -0.014 | -0.052, 0.024 | 0.48 |
| LDL | -0.001 | -0.01, 0.007 | 0.76 |
| eGFR | 0.005 | -0.01, 0.021 | 0.51 |
| hsCRP (log) | 0.098 | -0.102, 0.298 | 0.34 |
| Education | 0.009 | -1.035, 1.052 | 0.99 |
| Marijuana use | 0.3 | -0.552, 1.153 | 0.49 |
| Current cocaine use | 0.362 | -0.739, 1.463 | 0.52 |
| WHR | 0.746 | -3.442, 4.935 | 0.73 |
| rs2954029 (*TRIB1*, intergenic) |  |  |  |
| Current cigarette smoking | -0.023 | -0.398, 0.351 | 0.90 |
| BMI | 0.004 | -0.019, 0.027 | 0.74 |
| LDL | -0.002 | -0.007, 0.003 | 0.53 |
| eGFR | 0.002 | -0.008, 0.012 | 0.73 |
| hsCRP (log) | -0.111 | -0.233, 0.012 | 0.08 |
| Education | 0.153 | -0.539, 0.845 | 0.66 |
| Marijuana use | -0.102 | -0.617, 0.414 | 0.70 |
| Current cocaine use | 0.082 | -0.671, 0.835 | 0.83 |
| WHR | 0.498 | -2.022, 3.018 | 0.70 |
| rs4911241 (*NOL4L*, intron) |  |  |  |
| Current cigarette smoking | 0.261 | -0.221, 0.743 | 0.29 |
| BMI | 0.001 | -0.029, 0.031 | 0.94 |
| LDL | -0.0002 | -0.006, 0.006 | 0.95 |
| eGFR | -0.0001 | -0.013, 0.013 | 0.98 |
| **hsCRP (log)** | **-0.182** | **-0.348, -0.015** | **0.03** |
| Education | 0.7 | -0.209, 1.608 | 0.13 |
| Marijuana use | -0.19 | -0.906, 0.525 | 0.60 |
| Current cocaine use | 0.048 | -0.97, 1.066 | 0.93 |
| WHR | -0.202 | -3.189, 2.785 | 0.89 |
| *BMI*, body mass index; *CI*, confidence interval; *hsCRP*, high-sensitivity C-reactive protein; *eGFR*, estimated glomerular filtration rate; *WHR*, waist-hip ratio. | | | |
| ^a^Adjusted for age, sex, and first five principal components. | | | |
